# Supplementary material for: Food Allergy Education and Management in Schools: A Scoping Review on Current Practices and Gaps
Source: Nutrients. 2022 Feb 9;14(4):732. doi: 10.3390/nu14040732 (PMC8879822; doi:10.3390/nu14040732)
Supplement: Supplementary file 1 [file nutrients-14-00732-s001.zip › nutrients-1573383-supplementary.pdf]

Table S1. Search strategy

Number	Search Terms
1	Food hypersensitivity/
2	(food hypersensitivit* or food intoleran* or food allerg* or food hyper sensitivit*).tw,kw.
3	1 or 2
4	Schools/ or schools, nursery/
5	Exp Child Day Care Centers/
6	(school* or daycare* or day care* or preschool*).tw,kw.
7	4 or 5 or 6
8	3 and 7
	OVID-MedLine (n = 535)<br>PsycINFO (n = 61)<br>Scopus (n = 1,414)<br><br>Total results: (N = 2,010)
